# 肺腺癌中m6A RNA甲基化修饰特征与免疫微环境相关性分析

**DOI:** 10.3779/j.issn.1009-3419.2022.103.02

**Published:** 2022-05-20

**Authors:** 冀 柯, 健 崔, 兴国 杨, 鑫 杜, 波波 马, 磊 于

**Affiliations:** 1 100730 北京，首都医科大学附属北京同仁医院胸外科 Department of Thoracic Surgery, Beijing Tongren Hospital, Capital Medical University, Beijing 100730, China; 2 100068 北京，首都医科大学康复医学院，中国康复研究中心 School of Rehabilitation Medicine, Capital Medical University, China Rehabilitation Research Center, Beijing 100068, China

**Keywords:** 肺腺癌, m6A修饰, 免疫微环境, LRPPRC, Lung adenocarcinoma, m6A modification, Immune microenvironment, LRPPRC

## Abstract

**背景与目的:**

m6A RNA甲基化修饰在肺癌的发生与进展中起着重要作用，可以调节肿瘤免疫进而影响疾病预后。目前很多集中在某些特定的m6A效应器的差异表达及对肿瘤免疫细胞浸润的影响，但单个效应器表达的变化远远不足以反映m6A修饰特征的全貌，且关于m6A修饰对肺腺癌免疫微环境影响的研究仍较少。本研究拟探讨不同m6A修饰模式对肺腺癌中免疫微环境的影响。

**方法:**

从癌症基因组图谱数据库（The Cancer Genome Atlas, TCGA）、加州大学圣克鲁兹分校泛癌全基因数据分析工具数据库（University of California Santa Cruz Xena, UCSC Xena）、基因表达综合数据库（Gene Expression Omnibus, GEO）获取肺腺癌相关数据信息。使用Maftools R分析肺腺癌队列中24个m6A效应器的基因突变，比较肺腺癌组织和正常组织中m6A效应器的表达差异，并通过*Cox*回归分析进行生存分析。通过Consensus Cluster Plus R非监督聚类的方法构建m6A修饰模式，进行m6A聚类生存分析、GSVA通路富集分析、免疫评分及免疫细胞浸润分析。在68例肺腺癌组织中，通过免疫组化分析LRPPRC蛋白表达水平与CD8、CD68的表达水平，验证LRPPRC与CD8^+^细胞毒性T细胞及巨噬细胞浸润的关系。

**结果:**

在567例肺腺癌样本中有150例发生了m6A效应器突变，频率为26.46%。与正常组织相比，肺腺癌组织中共有6个读取器和3个写入器的表达明显上调。IGF2BP1和HNRNPC是影响肺腺癌患者预后的独立危险因素，且各效应器之间也存在大量的相互作用。构建了3种具有不同免疫细胞浸润特征和临床预后的m6A修饰模式。发现LRPPRC的表达与包括杀伤性T细胞和巨噬细胞在内的多种免疫细胞的浸润呈负相关，并在68例肺腺癌组织中得到验证。

**结论:**

m6A修饰对肺腺癌免疫微环境的调节起着重要作用，LRPPRC有可能作为预测抗PD1免疫治疗效果的潜在生物标记物。

## Abstract (en)

**Background and objective:**

m6A RNA methylation modification plays an important role in the occurrence and progression of lung cancer and regulates tumor immunity. Current studies mostly focus on the differential expression of some specific m6A effectors and infiltrating immune cell. m6A methylation modification is the result of mutual adjustment and balance between effectors, and changes in the expression of one or two effectors are far from enough to reflect the panorama of m6A methylation. The role of m6A in the immune microenvironment of lung adenocarcinoma (LUAD) is still poorly understood. The aim of this study is to investigate the effect of different m6A modification patterns in immune microenvironment of LUAD.

**Methods:**

LUAD data was obtained from The Cancer Genome Atlas (TCGA), University of California Santa Cruz Xena (UCSC Xena) and Gene Expression Omnibus (GEO) databases. Gene mutation, differential expression and survival analysis were performed for 24 m6A effectors. The m6A modification pattern was constructed by unsupervised clustering method, and the m6A clusters survival analysis, gene set variation analysis, immune score and immune cell infiltration analysis were performed. The association between LRPPRC protein expression levels and infiltration of CD8^+^ cytotoxic T lymphocytes and CD68^+^ macrophages in the tumor microenvironment was validated by immunohistochemistry in LUAD tissue microarray with 68 cases.

**Results:**

The mutations of m6A effector were found in 150 of 567 LUAD cases with a frequency of 26.46%. 6 readers and 3 writers were significantly up regulated in LUAD tissues compared with normal tissues. IGF2BP1 and HNRNPC are the independent risk factors for prognosis of LUAD. Abundant cross-talks among writers, erasers and readers were demonstrated. Three m6A modification patterns with different immune cell infiltration characteristics and clinical prognosis were established. Among m6A effectors, LRPPRC was found to be inversely associated with the infiltration of CD8^+^ cytotoxic T lymphocytes and CD68^+^ macrophages, and was validated in 68 LUAD tissues.

**Conclusion:**

m6A modification patterns play non-negligible roles in regulating the immune microenvironment. LRPPRC has potential to be a new biomarker for checkpoint inhibitor immunotherapy.

RNA修饰作为转录后调控的重要组成部分^[1]^，其相关的生物学功能也越来越引起了人们广泛的关注^[2-4]^。在这些修饰中，N6-甲基腺嘌呤（N6-methyladenosine, m6A），即位于核糖核酸N6位点的腺苷甲基化，广泛存在于mRNA和非编码RNA中。m6A修饰通过的其“效应器”发挥作用，包括甲基转移酶（“写入器”）、去甲基化酶（“擦除器”）和m6A修饰位点结合蛋白（“读取器”）^[5, 6]^，调节着RNA的稳定性、剪接、翻译以及出核，从而影响基因表达，在几乎所有重要的生物学过程中发挥作用^[7-9]^。研究^[10-13]^表明m6A效应器在多种肿瘤中异常表达，参与肿瘤的增殖、转移、分化、血管生成以及免疫逃逸。

肺腺癌长期以来一直采用传统的化疗、放疗和手术治疗^[14]^，随着靶向治疗和免疫治疗的突破从根本上改变了肺腺癌的治疗方式^[15]^，但只有小部分患者可以从免疫治疗中获益^[16]^。免疫治疗是肿瘤免疫系统的重建，它依赖于肿瘤细胞和浸润的免疫细胞之间的相互作用，依赖于免疫细胞在肿瘤微环境中的浸润程度^[17]^。某些m6A修饰在肺腺癌的进展中起着重要作用，但关于m6A修饰对肺腺癌免疫微环境影响的研究较少。因此，综合分析由多个m6A效应器在肿瘤微环境中的对免疫细胞浸润的影响，将有助于我们加深其对免疫微环境调节的认识，促进肺腺癌患者更好地受益于免疫治疗。在本研究中，我们选定了24个m6A效应器，分析了m6A修饰与肿瘤微环境的免疫细胞浸润的相关性，构建了三种具有不同免疫特征和临床预后的m6A修饰模式，并发现LRPPRC有可能作为预测免疫治疗效果的潜在生物标记物。

## 1 材料与方法

### 1.1 数据获取与处理

从癌症基因组图谱数据库（The Cancer Genome Atlas, TCGA）（https://portal.gdc.cancer.gov/）和加州大学圣克鲁兹分校泛癌全基因数据分析工具数据库（University of California Santa Cruz Xena, UCSC Xena）（https://xena.ucsc.edu/）下载肺腺癌队列的基因突变数据、拷贝数变异数据、RNA-seq数据和临床资料。从基因表达综合数据库（Gene Expression Omnibus, GEO）（http://www.ncbi.nlm.nih.gov/geo）下载基因芯片数据集（GSE40419、GSE41271、GSE72094和GSE126044）。GSE40419数据集包含87个肺腺癌肿瘤组织和77个相邻正常肺组织的RNA表达数据。GSE41271和GSE72094数据集分别包含178个和381个肺腺癌肿瘤组织的RNA表达数据，以及完整的临床和生存信息。GSE126044数据集包含16个非小细胞肺癌（non-small cell lung cancer, NSCLC）肿瘤组织的RNA表达数据和抗PD-1免疫治疗的应答信息。将数据进行log_2_转换及标准化处理。

### 1.2 m6A修饰基因突变、差异表达及生存分析

Maftools R软件包用于分析TCGA肺腺癌队列中24个m6A效应器的基因突变。采用Student *t*检验比较TCGA肺腺癌队列和GSE40419队列中肿瘤组织和邻近正常组织中24种m6A效应器的差异表达。使用单变量*Cox*回归分析在m6A效应器和临床表型之间进行生存分析，然后进行多变量*Cox*回归分析，从而确定影响肺腺癌患者预后的独立危险因素。*Spearman*相关性分析用于评估不同m6A效应器之间的相关性。

### 1.3 m6A修饰模式的聚类、富集及免疫浸润分析

为了确定肺腺癌患者中由24个m6A效应器调节的m6A修饰模式，使用Consensus Cluster Plus R软件包进行非监督聚类分析，评估TCGA肺腺癌队列中m6A修饰特征。通过GSVA R包进行基因集变异分析，使用“C2Broadset”的KEGG基因集，阐明不同m6A聚类之间的不同生物学过程。通过CIBERSORT评估不同m6A修饰模式下的免疫细胞成分。根据Charoentong的研究^[18]^，使用GSVA R包中ssGSEA单样本基因集富集分析的方法计算免疫浸润细胞的相对丰度。Estimate R软件包用于计算肺腺癌患者的基质和免疫评分。

### 1.4 免疫组织化学分析

肺腺癌样本的组织芯片购于SHANGHAI OUTDO BIOTECH（HLugA150CS03），参照此前的研究方法^[19]^进行免疫组化分析。使用LRPPRC抗体（Abcam #ab97505）分析LRPPRC的蛋白表达水平。分别采用CD8（ZSGB-BIO #ZA-0508）和CD68（Abcam #125212）的表达水平来评估CD8^+^细胞毒性T细胞及巨噬细胞。免疫组化评分=染色面积（< 30%为1分，30%-60%为2分， > 60%为3分）×染色强度（无表达为0，弱阳性为1分，中阳性为2分，强阳性为3分）。根据免疫组化评分，分为低表达组（0分-3分）、中表达组（4分-5分）和高表达组（6分-9分）。

## 2 结果

### 2.1 肺腺癌中m6A修饰的突变和表达差异

本研究共纳入了24个m6A效应器进行分析，其中包括10个写入器，2个擦除器，12个读取器（表 1）。我们首先总结了m6A效应器在基因组水平上的变异。567例肺腺癌样本中有150例至少发生一个在m6A效应器突变，频率为26.46%，其中*ZC3H13*的突变频率最高，频率为4%，其次是*KIAA1429*和*IGF2BP1*（图 1A）。相关分析发现，写入器*METTL5*和*RBM15*的突变分别与读取器*ELAVL1*和*IGF2BP1*的突变显著相关（*P* < 0.05）（图 1B）。拷贝数变异（copy number variation, CNV）分析表明，CNV变异在m6A效应器中普遍存在（图 1C），除了ELAVL1和YTHDC2，大多数读取器的CNV是扩增的（图 1C）。

**表 1:** 24个m6A效应器列表 Information of 24 m6A effectors

Gene symbol	Full name	m6A effector
*METTL3*	N6-adenosine-methyltransferase catalytic subunit	Writer
*METTL5*	rRNA N6-adenosine-methyltransferase METTL5	Writer
*METTL14*	N6-adenosine-methyltransferase non-catalytic subunit	Writer
*METTL16*	RNA N6-adenosine-methyltransferase METTL16	Writer
*WTAP*	Pre-mRNA-splicing regulator WTAP	Writer
*RBM15*	RNA-binding protein 15	Writer
*RBM15B*	Putative RNA-binding protein 15B	Writer
*KIAA1429*	Protein virilizer homolog	Writer
*ZC3H13*	Zinc finger CCCH domain-containing protein 13	Writer
*CBLL1*	E3 ubiquitin-protein ligase Hakai	Writer
*ZCCHC4*	rRNA N6-adenosine-methyltransferase ZCCHC4	Writer
*FTO*	Alpha-ketoglutarate-dependent dioxygenase FTO	Eraser
*ALKBH5*	RNA demethylase ALKBH5	Eraser
*YTHDF1*	YTH domain-containing family protein 1	Reader
*YTHDF2*	YTH domain-containing family protein 2	Reader
*YTHDF3*	YTH domain-containing family protein 3	Reader
*IGF2BP1*	Insulin-like growth factor 2 mRNA-binding protein 1	Reader
*HNRNPA2B1*	Heterogeneous nuclear ribonucleoproteins A2/B1	Reader
*FMR1*	Synaptic functional regulator FMR1	Reader
*LRPPRC*	Leucine-rich PPR motif-containing protein, mitochondrial	Reader
*HNRNPC*	Heterogeneous nuclear ribonucleoproteins C1/C2	Reader
*YTHDC1*	YTH domain-containing protein 1	Reader
*YTHDC2*	YTH domain-containing protein 2	Reader
*ELAVL1*	ELAV-like protein 1	Reader

**图 1:**
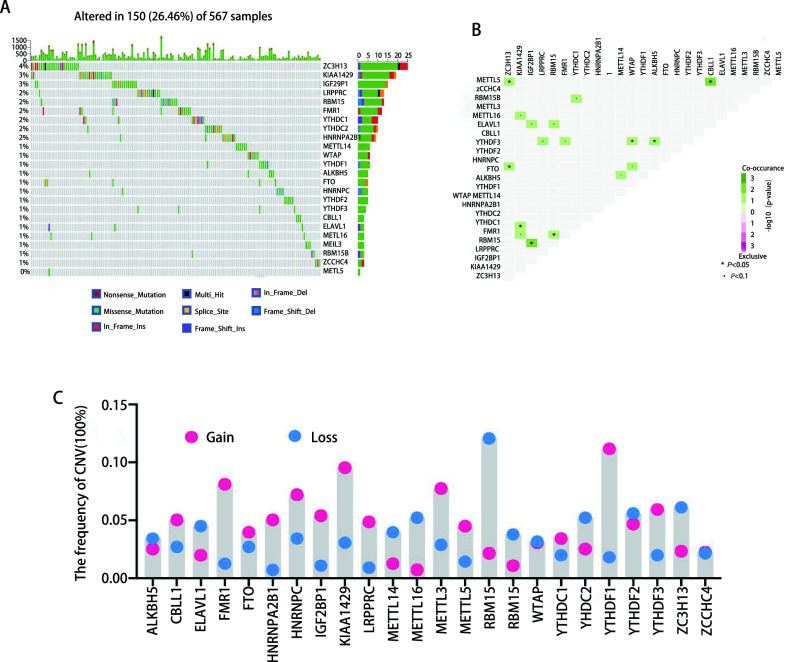
肺腺癌中m6A效应器的突变、共现分析及拷贝数变异。A：24个m6A效应器在567例TCGA-肺腺癌队列中突变频率；B：m6A效应器的共现分析；C：m6A效应器的拷贝数变异。 Mutation, co-occurrence analysis and copy number variation of m6A effector in lung adenocarcinoma. A: The mutation frequency of 24 m6A effectors in 567 patients with LUAD in TCGA-LUAD data sets; B: The co-occurrence of mutations between different m6A effectors; C: CNV of each m6A effectors. TCGA: The Cancer Genome Atlas; LUAD: Lung adenocarcinoma; CNV: copy number variation.

为了研究m6A效应器在转录水平上的变化，我们分别比较了TCGA肺腺癌队列和GSE40419队列中肿瘤组织与癌旁正常组织之间m6A效应器的mRNA表达水平（图 2A和图 2B）。与正常组织相比，癌组织中共有6个读取器（HNRNPA2B1、HNRNPC、IGF2BP1、LRPPRC、YTHDF1、YTHDF2）和3个写入器（KIAA1429、METTL5、RBM15）的表达明显上调；相反，癌组织中唯一下调的是擦除器FTO（图 2C和图 2D），表明m6A修饰可能在肺腺癌中具有促癌作用。

**图 2:**
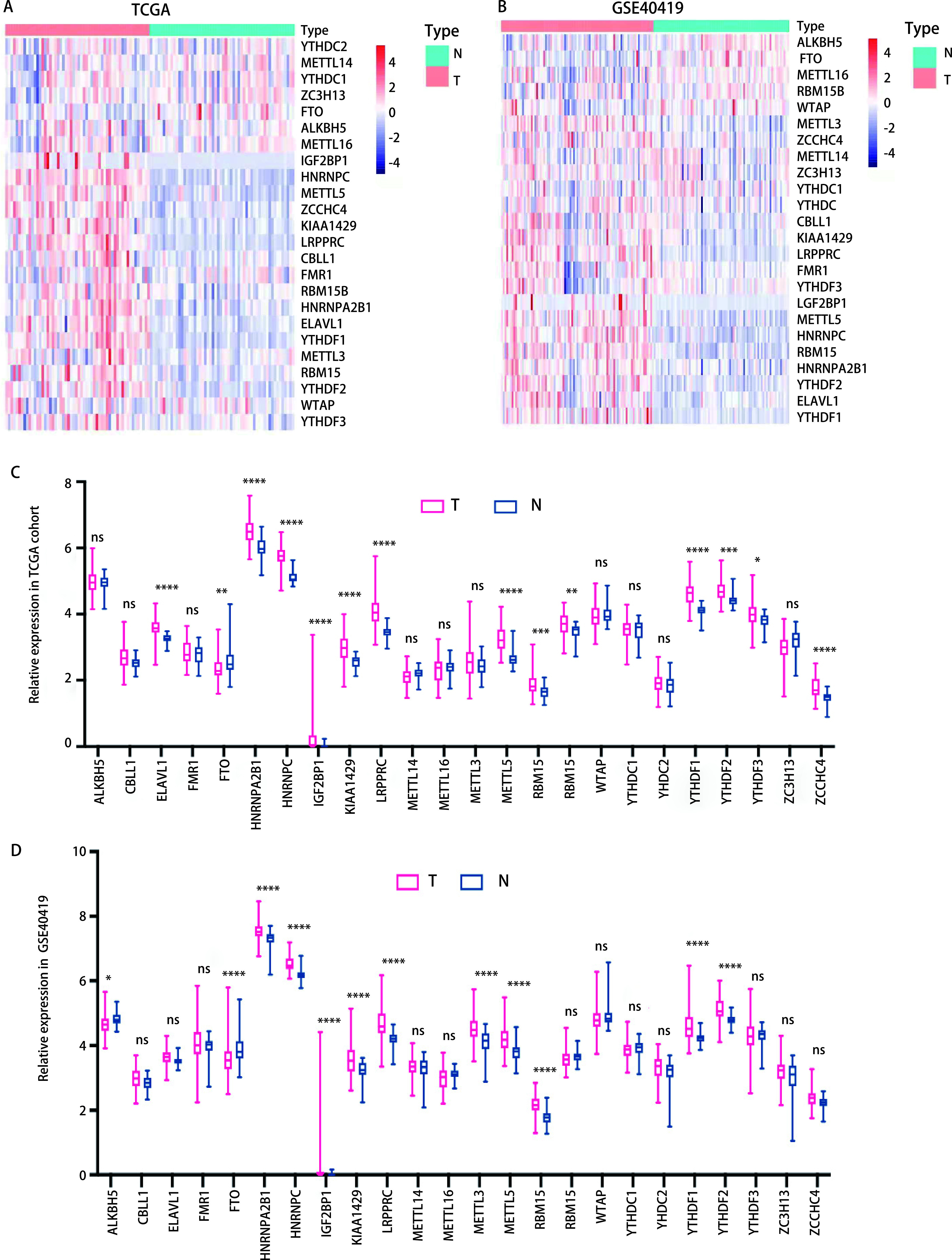
肺腺癌组织与癌旁正常组织m6A效应器的表达。A、B：TCGA-肺腺癌队列（A）和GSE40419队列（B）中肺腺癌组织与正常组织m6A效应器的表达热图；C、D：TCGA-肺腺癌队列（C）和GSE40419队列（D）中肺腺癌组织与正常组织m6A效应器的表达水平。T：肿瘤组织；N：正常组织；ns：无统计学差异；^*^P < 0.05；^**^P < 0.01；^***^P < 0.001；^****^P < 0.000, 1。 Expression of m6A effector in lung adenocarcinoma tissue and adjacent normal tissue. A and B: Heat map for the expression levels of m6A effectors in LUAD and paired normal lung tissues of TCGA-LUAD cohort (A) and GSE40419 cohort (B); C and D: The expression level of m6A effectors in tumor tissues and paired normal lung tissues of TCGA-LUAD (C) and GSE40419 (D) cohorts. T: tumor; N: normal; ns: no statistical significance; ^*^P < 0.05; ^**^P < 0.01; ^***^P < 0.001; ^****^P < 0.000, 1.

### 2.2 m6A效应器对肺腺癌预后的影响及相互作用

本研究通过单变量*Cox*回归分析，分析了24个m6A效应器的表达异常对肺腺癌患者预后的影响。结果发现，METTL5、HNRNPA2B1、HNRNPC和IGF2BP1的高表达与不良预后显著相关（*P* < 0.05）。考虑到临床病理特征对患者生存率的影响，纳入患者性别、年龄和肿瘤原发灶-淋巴结-转移（tumor-node-metastasis, TNM）分期进行多变量*Cox*回归分析，结果显示IGF2BP1和HNRNPC是影响肺腺癌患者预后的独立危险因素（图 3）。

**图 3:**
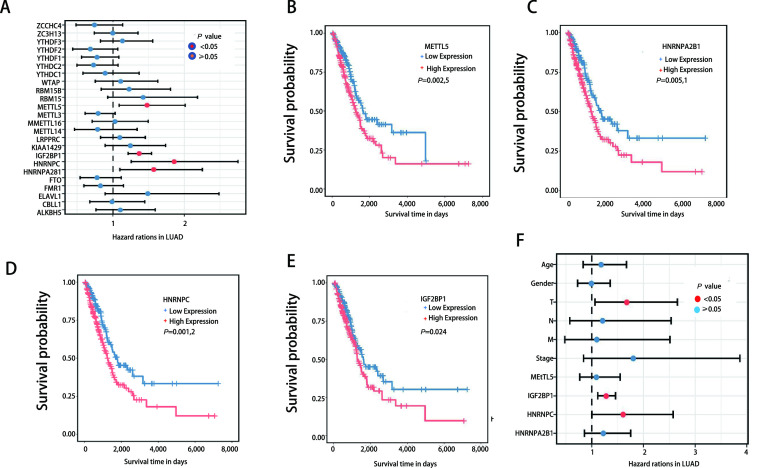
m6A效应器对肺腺癌预后的影响。A：TCGA-肺腺癌队列中24个m6A效应器的单变量*Cox*回归分析；B-E：METT5（B）、HNRNPA2B1（C）、HNRNPC（D）和IGF2BP1（E）的*Kaplan-Meier*生存曲线；F：临床病理学特征和上述4个效应器的多变量*Cox*回归分析。 The prognostic value of m6A effectors in LUAD. A: The forest plot of the univariate *Cox* regression analysis of 24 m6A effectors in TCGA-LUAD cohorts; B-E: The *Kaplan-Meier* curves of METT5 (B), HNRNPA2B1 (C), HNRNPC (D) and IGF2BP1 (E); F: The forest plot of the multivariate *Cox* regression analysis of the clinicopathological characteristics and the above four effectors.

m6A修饰是甲基化和去甲基化的动态平衡过程，受效应器的表达和活性的影响。因此，我们在m6A效应器之间进行*Spearman*相关性分析，以进一步发现不同m6A效应器之间的相互作用。发现某些写入器和读取器之间存在显著的正相关关系，如KIAA1429和YTHDF3、METTL14和YTHDC1、ZC3H13和YTHDC1、KIAA1429和LRRPRC（图 4A-图 4D）。部分写入器也表现出显著的正相关，如KIAA1429和CBLL1、METTL14和ZC3H13，这反映了它们的功能协同作用（图 4E、图 4F）。

**图 4:**
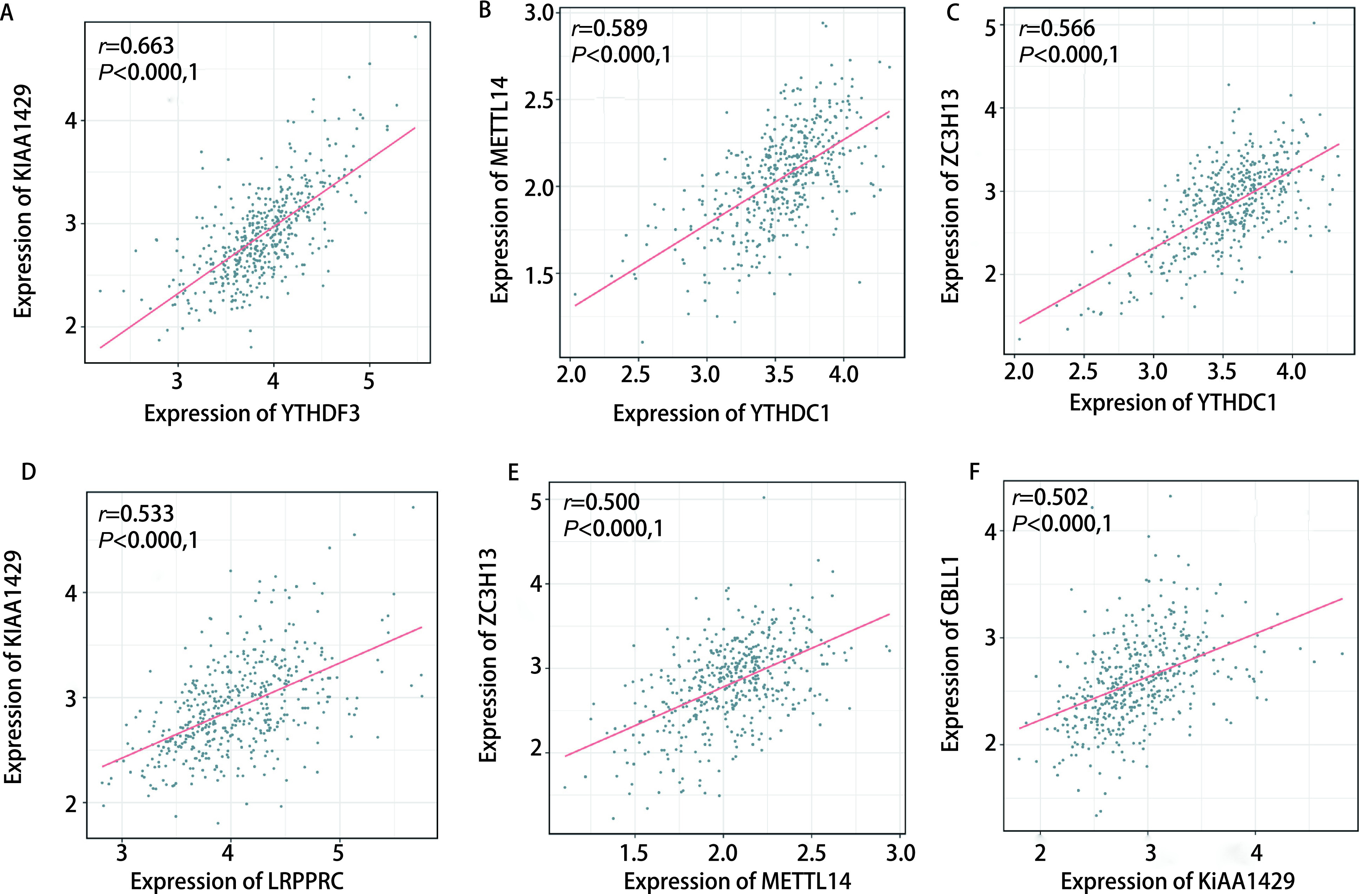
肺腺癌m6A效应器间相关性分析。A：KIAA1429和YTHDF3；B：METTL14和YTHDC1；C：ZC3H13和YTHDC1；D：KIAA1429和LRPPRC；E：CBLL1和KIAA1429；F：ZC3H13和METTL14。 Correlation analysis between m6A effectors in lung adenocarcinoma. A: KIAA1429 and YTHDF3; B: METTL14 and YTHDC1; C: ZC3H13 and YTHDC1; D: KIAA1429 and LRPPRC; E: CBLL1 and KIAA1429; F: ZC3H13 and METTL14.

### 2.3 m6A修饰模式及相关免疫特征

为了识别肺腺癌中不同的m6A修饰模式，我们根据24个m6A效应器的表达水平进行非监督聚类分析，从而确定三种不同的m6A修饰模式，称为m6A聚类A、B、C。对三组m6A聚类患者的预后分析，发现三组患者的总体存活率有显著差异，其中m6A聚类A的预后最好（图 5A）。GSVA富集分析结果表明，m6A聚类A富集了较多与免疫激活相关的信号通路，如趋化因子信号通路、T细胞受体信号通路和B细胞受体信号通路等（图 5B）；而m6A聚类B则与致癌信号通路的激活相关，包括DNA复制、细胞周期及三羧酸循环等（图 5C）。通过比较不同m6A聚类之间的免疫评分和基质评分，发现m6A聚类A的基质评分和免疫评分最高（图 5D）。免疫细胞浸润相对丰度计算结果显示，m6A聚类A在肿瘤微环境中有更多的免疫细胞浸润，如活化B细胞、巨噬细胞、骨髓源抑制细胞、自然杀伤细胞、T1辅助细胞和T2辅助细胞（图 5E）。

**图 5:**
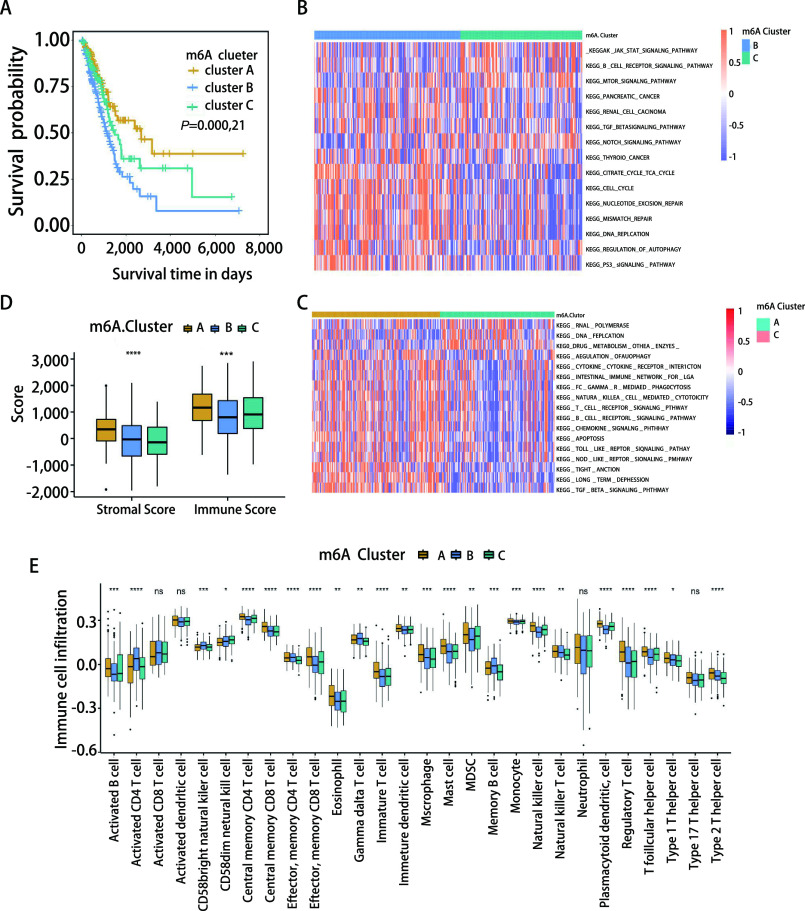
不同m6A修饰模式的生存分析、富集通路及免疫浸润分析。A：m6A聚类的生存分析；B-C：不同m6A修饰模式中的GSVA富集分析；D：m6A聚类的基质评分和免疫评分；E：m6A聚类中28种免疫细胞的浸润丰度。^*^P < 0.05；^**^P < 0.01；^***^P < 0.001；^****^P < 0.000, 1。 Survival analysis, enrichment pathway and immune infiltration analysis in distinct m6A modification pattern. A: Survival analyses of three m6A clusters; B-C: GSVA enrichment analysis in distinct m6A modification patterns; D: The stromal score and immune score of three m6A clusters; E: Abundance of the infiltration of 28 immune cells in the three m6A clusters. ^*^P < 0.05; ^**^P < 0.01; ^***^P < 0.001; ^****^P < 0.000, 1.

### 2.4 m6A效应器的表达与免疫细胞浸润

由于m6A修饰模式影响了肿瘤微环境中的免疫细胞浸润，我们通过*Spearman*相关性分析进一步研究了24个m6A效应器的表达与肿瘤微环境中免疫细胞浸润程度的相关性（图 6A）。发现读取器LRPPRC与免疫细胞浸润程度呈负相关。通过对LRPPRC表达进行进一步的分组研究，结果表明，与LRPPRC低表达组相比，LRPPRC高表达组的基质评分和免疫评分均较低（图 6B）。信号通路富集分析显示，LRPPRC低表达组的免疫激活通路明显富集（图 6C）。我们进一步比较了28种免疫细胞的浸润情况，发现其中24种在LRPPRC低表达组的肿瘤组织中浸润更多，特别是树突细胞、CD8^+^细胞毒性T淋巴细胞以及巨噬细胞（图 6D）。同时，在LRPPRC低表达组的肿瘤细胞中树突细胞相关共刺激因子和黏附因子的表达也显著上调（图 6E）。我们进一步评估了LRPPRC对肺腺癌患者（GSE126044）抗程序性死亡受体1（programmed death 1, PD-1）免疫治疗应答率的影响，发现在8例LRPPRC高表达患者中仅1例应答，而在8例LRPPRC低表达患者中发现4例应答（图 6F）。因此，我们推测LRPPRC的表达可能通过抑制肿瘤微环境中免疫细胞的浸润和激活，从而影响了免疫治疗的应答，是一种潜在的免疫治疗标记物。

**图 6:**
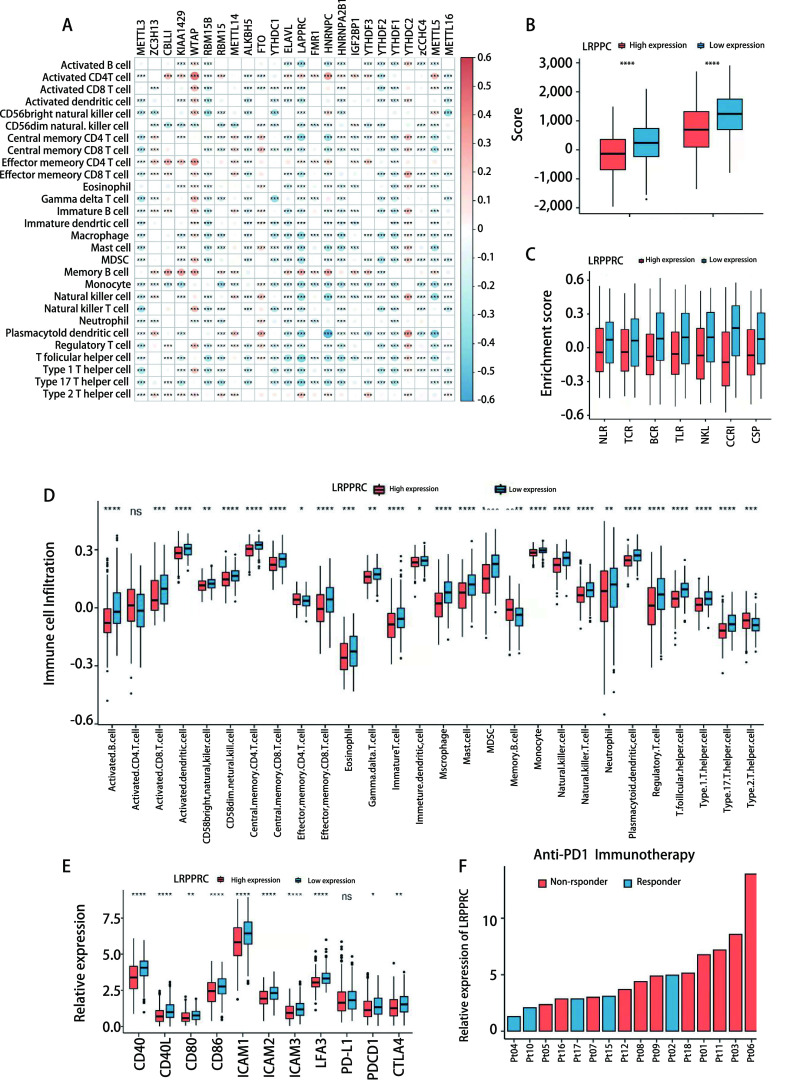
免疫细胞浸润与m6A效应子的相关性分析。A：m6A效应器表达与免疫细胞浸润的相关性分析热图；B：LRPPRC高、低表达组的基质评分和免疫评分；C：LRPPRC高、低表达组免疫相关信号通路的富集评分；D：LRPPRC高、低表达组的免疫细胞浸润情况；E：树突细胞相关共刺激因子和黏附因子在LRPPRC高、低表达组间的表达水平；F：肺腺癌患者LRPPRC的相对表达水平与对抗PD1免疫治疗的应答。ns：无统计学差异；^*^P < 0.05；^**^P < 0.01；^***^P < 0.001；^****^P < 0.000, 1。 Correlation analysis of the infiltration of immune cells and each m6A effector. A: Heatmap of the spearman correlation coefficients between immune cells infiltration and the expression level of m6A effectors; B: Stromal and immune score between LRPPRC high and low expression group; C: The enrichment score of immune associated signal pathways between LRPPRC high and low expression group; D: The infiltration of immune cells in LRPPRC high and low expression group; E: The expression level of DCs-related costimulatory factors and adhesion factors on tumor cells with LRPPRC high or low expression; F: The relative expression level of LRPPRC and response to anti-PD1 immunotherapy of LUAD. ns: no statistical significance; ^*^P < 0.05; ^**^P < 0.01; ^***^P < 0.001; ^****^P < 0.000, 1.

### 2.5 LRPPRC在组织标本中的表达与免疫细胞浸润

采用免疫组化的方法，在68例肺腺癌组织中验证了LRPPRC的表达与CD8^+^细胞毒性T细胞及巨噬细胞浸润的相关性。结果表明，LRPPRC的表达与CD8^+^细胞毒性T淋巴细胞和巨噬细胞的浸润呈负相关。在LRPPRC高表达患者中，肿瘤微环境中的CD8^+^细胞毒性T淋巴细胞和巨噬细胞浸润显著增加（*P* < 0.001）；相反，LRPPRC高表达患者的免疫细胞浸润程度降低（*P* < 0.001）（图 7）。验证结果与此前分析结论相一致。

**图 7:**
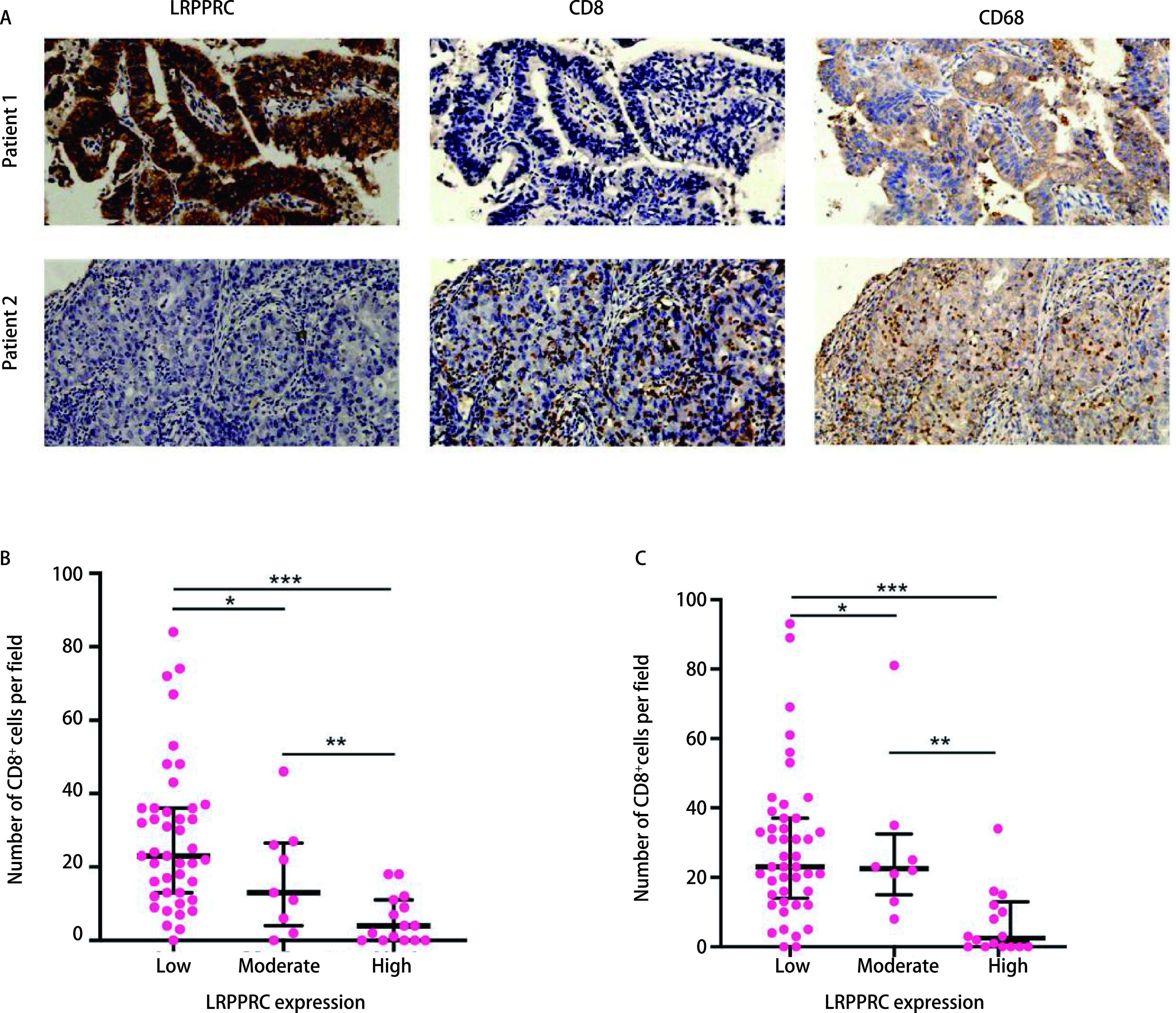
免疫组化分析肿瘤微环境中LRPPRC表达、CD8^+^细胞毒性T细胞和巨噬细胞浸润。A：肺腺癌组织中LRPPRC、CD8和CD68的免疫组织化学染色（原始放大倍数，x100）；B-C：根据LRPPRC表达分组，每视野CD8^+^细胞毒性T细胞（B）和CD68^+^巨噬细胞（C）的浸润数量。^*^P < 0.05; ^**^P < 0.01; ^***^P < 0.001。 Analyses of LRPPRC expression, CD8^+^ cytotoxic T lymphocytes and CD68^+^ macrophages infiltration in TME by immunohistochemistry. A: Immunohistochemical staining of LRPPRC, CD8 and CD68 in LUAD tissues (original magnification, x100); B-C: The number of CD8^+^ cytotoxic T lymphocytes (B) and CD68^+^ macrophage (C) infiltration per filed was calculated in different group according to LRPPRC expression with 68 LUAD patients. ^*^P < 0.05; ^**^P < 0.01; ^***^P < 0.001.

## 3 讨论

在本研究中，我们通过对肺腺癌中m6A修饰突变率的研究，以及对比肺腺癌组织与正常组织中m6A效应器在基因组和转录组层面的表达差异，发现了m6A效应器可能对肺腺癌的发生和发展起着重要作用。通过研究m6A效应器对肺腺癌患者预后的影响，发现m6A效应器对肺腺癌患者的预后有重要影响。同时，我们发现m6A写入器、擦除器和读取器之间的存在大量的相互作用，因此m6A效应器的生物学功能可能是各效应器的共同作用而实现的，即通过不同的m6A修饰模式而非单个基因改变。

近年来，已有研究^[20]^聚焦在不同肿瘤中m6A效应器与免疫相关的关系，发现了在很多肿瘤中m6A效应器与免疫功能间也存在着一定的相互作用。在本研究中，我们不仅研究了某个特定m6A效应器对肺腺癌免疫细胞浸润的影响，而且从宏观层面全面分析了不同m6A修饰模式对肺腺癌免疫微环境的调控。我们基于24个m6A效应器的非监督聚类分析构建了三种不同的m6A修饰模式。其中m6A聚类A的预后最好。在m6A聚类A中，YTHDC2、METT14、ZC3H13、FTO和ALKBH5显著高表达，这些基因都与各种免疫细胞浸润呈正相关。富集分析也提示m6A聚类A富集了较多与免疫激活相关的信号通路。m6A聚类A表现出大量的免疫浸润，从而有利于肿瘤对免疫治疗应答，这也提示预后较好的原因之一。与m6A聚类A相比，m6A聚类B中的免疫细胞浸润显著减少，而DNA复制、细胞周期、p53信号通路和其他肿瘤进展相关通路明显富集。同时，LRPPRC、IGF2BP1、METTL5和HNRNPC在m6A聚类B中高表达。已有研究^[16, 21-24]^表明，这些基因在各类肿瘤中都广泛参与了促使肿瘤进展的相关生物学过程，并与导致生存期缩短相关。这可能也是m6A聚类B临床预后最差的原因之一。总之，在不同m6A聚类之间，免疫微环境状态不同且富集的相关通路不同，都表明不同的m6A修饰模式对肺腺癌发生、发展及预后起着重要作用。

LRPPRC作为m6A的读取效应器，因其在肺腺癌中的显著高表达且与大量免疫细胞在肿瘤微环境中的浸润呈负相关，这引起我们的关注。越来越多的证据^[25-27]^表明，免疫治疗的关键是有更多的免疫细胞浸入肿瘤。在我们的研究中，发现LRPPRC的高表达，减少了免疫细胞对肿瘤的浸润，从而降低了对抗PD1免疫治疗的敏感性。这也表明LRPPRC有可能作为抗PD1免疫治疗效果的生物标记物，并作为与免疫检查点抑制剂结合的治疗靶点。

总之，m6A修饰是一个复杂的转录后调控过程，需要多个效应器之间的共同作用。通过对m6A效应器的综合分析，我们发现不同的m6A修饰模式在免疫微环境的调节中起着重要作用，加深了我们对m6A修饰在肺腺癌中的作用及其机制的理解。同时，LRPPRC有可能作为预测抗PD1免疫治疗效果的潜在生物标记物，有待今后更加深入的研究。
